# Explaining household socio-economic related child health inequalities using multiple methods in three diverse settings in South Africa

**DOI:** 10.1186/1475-9276-10-13

**Published:** 2011-04-04

**Authors:** Lungiswa L Nkonki, Mickey Chopra, Tanya M Doherty, Debra Jackson, Bjarne Robberstad

**Affiliations:** 1 Health Systems Research Unit, Medical Research Council, Cape Town, South Africa; 2 Centre for International Health, University of Bergen, Norway; 3 UNICEF, New York, USA; 4 School of Public Health, University of the Western Cape, Cape Town, South Africa

## Abstract

**Background:**

Despite free healthcare to pregnant women and children under the age of six, access to healthcare has failed to secure better child health outcomes amongst all children of the country. There is growing evidence of socioeconomic gradient on child health outcomes

**Methods:**

The objectives of this study were to measure inequalities in child mortality, HIV transmission and vaccination coverage within a cohort of infants in South Africa. We also used the decomposition technique to identify the factors that contribute to the inequalities in these three child health outcomes. We used data from a prospective cohort study of mother-child pairs in three sites in South African. A relative index of household socio-economic status was developed using principal component analysis. This paper uses the concentration index to summarise inequalities in child mortality, HIV transmission and vaccination coverage.

**Results:**

We observed disparities in the availability of infrastructure between least poor and most poor families, and inequalities in all measured child health outcomes. Overall, 75 (8.5%) infants died between birth and 36 weeks. Infant mortality and HIV transmission was higher among the poorest families within the sample. Immunisation coverage was higher among the least poor. The inequalities were mainly due to the area of residence and socio-economic position.

**Conclusion:**

This study provides evidence that socio-economic inequalities are highly prevalent within the relatively poor black population. Poor socio-economic position exposes infants to ill health. In addition, the use of immunisation services was lower in the poor households. These inequalities need to be explicitly addressed in future programme planning to improve child health for all South Africans.

## Background

South Africa is the most consistently unequal economy in the world; the Gini coefficient (measure of income inequality) has increased from 0.64 in 1994 to 0.72 in 2005[1]. These inequalities are most obvious in child health outcomes [2] Evidence suggests that unless equality is explicitly measured and addressed, public health interventions tend to perpetuate existing inequalities [3]. Moser, Leon & Gwatkin [4] have pointed out that Millennium Development Goals (MDGs) could be reached globally while child health inequalities increase.

The post-apartheid period in South Africa has been bitter sweet. South Africa has experienced steady economic growth, and is respected globally for its constitution, which protects the humanity and dignity of all South Africans. Addressing inequalities has been a key priority since 1994[5]. During the apartheid era, the distribution of income was unbalanced and was strongly aligned with race. Even though racial inequality still exists, a new shift has occurred; disparities have widened amongst the black population [6]. There has also been rapid urbanisation, and consequently, an increase in urban slums and urban poverty. Therefore, relying solely on race and geography in order to analyse equality is becoming less useful.

Empirical analysis suggests that relative disparities have persisted and that infants and children under-5 years of age may actually be worse off than they were before 1994 in absolute terms [7-9], despite the increase in social spending on welfare, health, education and housing. In this situation, the challenge is how to measure and monitor progress, and to identify pathways and dynamics in child health inequalities. In an attempt to analyse the impact of interventions at a state level, local reports have separated child health outcomes by geographical areas and race [7]. However, these approaches assume that geographical areas and racial categories are homogeneous in socio-economic terms. We therefore need to research on health inequalities in South Africa, with parameters that better describe people's socio-economic conditions than geographical area and race. Furthermore, the 28% HIV prevalence in South Africa [10], means that we need, more knowledge in the areas of inequality in HIV/AIDS and child health.

There is little agreement on the best way to approach to measuring socio-economic position, and previous research studies have used other factors such as education, occupation, income and wealth[11].

In some instances researchers have used an asset index that combined a mixture of socio-economic indicators, which are hypothesised to interact with health outcomes in different ways, for instance income, and education [12,13]. Income can enable households to purchase health services; similarly, education can have a similar effect through its occupation effect (i.e. education can increase one's opportunity of getting employed and earning an income). Unlike income, education can facilitate a better understanding of health messages and thus increase the likelihood of the use of health services. Combining different factors such as these complicates the interpretation of findings and comparisons between studies become more challenging.

Compared to household income, the variables used to derive the asset indices are more easily observed and suffer less measurement and reporting error. Stewart & Simelane [11] compared the consistency of an asset index and an income variable in predicting child mortality at the household level and community level. They found both measures to be extremely similar, indicating that asset indexes can be valid measures of socio-economic position.

The aim of our study was to measure inequalities in child mortality, HIV transmission and vaccination coverage, and to identify the drivers of the inequality within a cohort of HIV positive and negative mother-child pairs in South Africa. These mother-child pairs were from three relatively poor communities. Our goal is that these analyses will be useful in informing South Africa's policy to reduce internal disparities in child health.

## Methods

### Measuring child health

Three child health outcomes were chosen: infant mortality (**<9 months)**, HIV transmission and immunisation coverage. Infant mortality was described as any death of an infant between birth and nine months of age (**<9 months)**. HIV transmission amongst infants was described as the number of HIV infected infants at 3, 24 or 36 weeks of age. Immunisation coverage was described as the number of infants who received complete immunisation (i.e. BCG, OPV3 and DTP3) at 24 weeks. The latter indicator is not only a child health indicator but also a health service use indicator. All three outcomes were binary variables.

### Measuring socio-economic position

Krieger, Williams and Moss [14] use the term socio-economic position to refer to various components of economic and social well-being, as related to class position. They argue that the commonly used term 'socio-economic status' blurs the distinction between two different aspects of 'socio-economic position', namely actual resources and status, meaning prestige-or rank-related characteristics. In light of this argument we use the term 'socio-economic position' instead of 'socio-economic status'.

### Selection of asset variables

There is no best practice on how to select variables to proxy living standards. Researchers have used variables, such as access to utilities and infrastructure (for example sanitation facilities, electricity and sources of water), durable goods [15,16], ownership of live stock[17], ownership of land[12], and the ratio of the number of people to the number of rooms in a household [18]. The number of variables used in studies has ranged from 10[12] to 30[19]. However, some researchers have used parsimonious sets of variables [20-26]. These sets include only 3-4 variables, for instance radio, television, type of floor [20-26]or electricity in a home, piped water and high status occupation[22].

For this study, we collected information on durable asset ownership, access to utilities and infrastructure, food availability at 24 weeks postpartum, maternal education, and household income. We avoided combining socio-economic indicators that are hypothesised to interact with health outcomes in different ways. In deciding on which variables to include in the index, we chose variables that would not increase households' ability to purchase health services or increase their understanding of health messages. Therefore, we did not include maternal education, food availability and household income in the index. The variables we considered including in the asset index were: fridge, radio, TV, stove, telephone/mobile phone, car, and infrastructural variables, such as sources of water supply, type of toilet and main fuel used for cooking.

Including of infrastructural variables in an asset index aimed at measuring household wealth can be viewed as inappropriate since these variables are publicly provided and therefore depend on availability of infrastructure at the community level. The argument of whether the asset index is reflects household's wealth or community wealth depends on how the assets were financed. In this study, the source of finance for the assets is less relevant because we are interested in the relative advantage that having these items presents to a household, and not who paid for them. For instance a household of high income earners within an area that does not have running water, electricity and adequate sanitation would be subject to the same constraints as other households within the same community. In addition, they would be at a disadvantage compared to their counterparts within households in areas that do not have these publicly provided indicators. Therefore, a comprehensive asset index should acknowledge that infrastructural variables play a role in living standards.

Another disclaimer to the including infrastructural variables in the asset index is related to the health outcome of choice. Infrastructural variables have a direct relationship with infant mortality over and above their indirect relationship as measures of socio-economic position. Excluding these variables is expected to yield smaller inequalities in outcomes such as infant mortality [27].

In light of infrastructural community level variables having a direct relationship with infant mortality, we constructed three indices; the first index only included consumer durables. This index included a fridge, radio, TV, stove, telephone/mobile phone and car. We did not base this choice of items on any economic value of the items themselves; it was based on the availability of items, which were only indicators of socio economic position on the original data set. The second index comprised infrastructural variables. The third index was a combination of both Index 1 and Index 2.

We assigned variable weights for all three indices, using the method of principal components analysis (PCA) [15]. PCA can be used as a data reduction or classification tool. In this paper, we have it as a tool for summarising variability within a set of variables. This method describes the variation of a set of multivariate data in terms of a set of uncorrelated linear indices or components of the original variables. Each consecutive linear combination is derived so that it explains as much as possible the variation in the original data, while being uncorrelated with other linear combinations [17]. The components are ordered so that the first component explains the largest possible variation in the original data. The subsequent components are uncorrelated with the preceding component and explain additional but less variation than the first component. McKenzie[19]demonstrated that only the first principal component was necessary for measuring wealth. Therefore, we extracted only the first principal component in this study. The first linear combination of variables (the first principal component [*c*_1_]) contains the most information on the variation in the underlying set of variables. The x_ij _terms refer to variable i for household j, and the y_hi _terms refer to the factor loadings (linear coefficients) for component h and variable i.

The first linear combination is:(1)

Using the first principal component, the percentage of variance were 44%, 69% and 42% for the consumer durables index (Index1), the index with infrastructural variables (Index2) and the index that combined the first two indices respectively. Index 1 and 2 were short and consisted of more homogeneous sets of items. As a consequence, households were grouped together in a small number of distinct clusters (clumping). Hence, it was not possible to categorise households into to five wealth groups. This problem is not unique to this study, McKenzie [19] identified problems of clumping and truncation as a major challenge for PCA-based asset indices. Therefore, we chose Index 3 as the measure of socio-economic position, since it included a range of asset variables that were broad enough to avoid clumping problems.

### Reliability of the asset index

We assessed the reliability of the asset index in two dimensions. Firstly we assessed whether the asset index produced clean separations across the least poor to the most poor for assets that are indicative of least poor and assets that are indicative of most poor. Secondly, we assessed the relationship between education, income and the food inventory index, and the asset index using cross-tabulations. We were interested in observing whether the asset index agreed with other measures of socio-economic position.

### Other measures of socio-economic position

We measured maternal education as the last standard passed at school. We measured the standard in terms of three categorical variables: no education and primary education, higher primary education, and successfully completed matric. We defined household income as the total household monthly income, including all sources of income. We did not collect information on household size.

We based the food inventory index on food items directly observed (by field researchers) in the households at 24 weeks after delivery. Similarly to the asset index variables, we assigned weights using PCA. We did not measure the quantities of these food items, and despite this limitation, observing that these items were present avoids the recall bias inherent in recall methods. For instance, it has been observed that households that have experienced an adverse child health outcome (such as death) have a heightened awareness of all the events prior to the death of the child compared to households who have not experienced that outcome. Similar observations have been made in households that have experienced severe hunger compared to those who have not. Nevertheless, this is a crude approximation of consumption and should be interpreted with caution.

### Measuring inequality

We used two different measures to explore the presence of inequality. Firstly, we divided the first principal component into quintiles so that each household was classified as most poor, poor, less poor or least poor, in terms of socio-economic position, with mean scores (i.e. first principal component) of -2.55, -1.50, -0.05, 1.37 and 2.73 respectively. Secondly, we use the concentration index to further explore drivers of inequality. Presenting findings using quintiles is common practice, especially in public health journals. This method is easier to understand since the study population is categorised into five equal groups representing the least poor to the most poor.

### The concentration index

The concentration index quantifies the degree of socioeconomic related inequality in a health variable [28].(2)

Where:

y*_i _*is the health variable of interest for the *i*th person;

μ is the mean of y;

*R*_*i *_is the *ith-*ranked individual in the socioeconomic distribution from the most disadvantaged (i.e. poorest) to the least disadvantaged (i.e., richest);

*n *is the number of persons

Unlike quintiles, the concentration index reflects the experiences of the entire population. Another advantage of the concentration index is that it is sensitive to the changes in size of the various groups, even if their health outcome mean has not changed [29]. The bounds of the concentration index are -1 to 1, where the sign indicates the direction of inequality, and the magnitude reflects both the strength of the relationship and the degree of variability in the health variable. The concentration index has been used in other studies to analyse inequalities in child health [30-32].

Using the asset index to measure socio-economic position presents a methodological challenge. The PCA has a mean of zero and takes negative values for about half of the households. Many measures of inequalities are divided by the mean and so do not apply to data that take negative values [33]. In order to address these challenges, we used the properties of the concentration index to rescale the index for the principal component analysis. This is explained in further detail below.

The concentration index depends on the relationship between the health variable and the rank of the living standard, and not on the variation in the living standard itself. The change in the living standard should therefore not affect the concentration index [34]. We rescaled the asset index by adding a constant of 3.0, which was the minimum whole number required to eliminate negative values. The range of the asset index prior to rescaling was -2.75 to 3.48. After rescaling the range was 0.25 to 6.48. This rescaling does not affect the contribution of each variable to the concentration index, since the rank ordering is unchanged. However, the relative magnitude of the elasticity and concentration index in the decomposition does change.

We calculated the concentration index using covariance and regression methods [34], and both yielded the same result. Inequalities are sometimes unavoidable, for example, there may be an unequal distribution due to biological factors or age. Alternatively, inequality adds a value judgement on the observed disparities; it often includes assessing whether the disparities are unjust, unfair and remediable.

### Decomposition of socio-economic inequality

Inequalities in child health outcomes are caused by inequalities in the factors that affect the variable of interest. Hence, an important policy question is what the relative contribution of each of these various inequalities in explaining child health outcomes inequality is. In order to address this question, we decomposed the concentration index. Wagstaff, van Doorslaer and Watanabe [32]} demonstrated that the concentration index of health can be expressed as the sum of contributions of various factors, together with an unexplained residual component. Together, the linear additive relationship between the health outcome variable *y_i_*, the intercept α, the relative contributions of *x_k_*determinants and the residual error ε*_i _*give the formula:(3)

Equation 4 demonstrates that the overall inequality in health outcome has two components: the explained component and the unexplained component. In the explained component, *β_k_*is the coefficient from a regression of health outcome on determinant *k*,  is the mean of determinant *k*, μ is the mean health outcome and *C_k _*is the concentration index for determinant *k*. In the unexplained component, *GC_ε_*is the generalised concentration index for the error term:(4)

The decomposition framework focuses on two main elements: the impact each determinant has on health outcomes  and the degree of unequal distribution of each determinant across income groups:(5)

The decomposition method was first introduced to be used with a linear, additively separable model. The child health outcomes in this study are non-linear. The two common choices common choices that yield probabilities in the range (0, 1) are the logit model and the probit model, both of which are fitted by maximum likelihood. One possibility when dealing with a discrete change from 0 to 1 is to use marginal or partial effects (d*y*/d*x*), which give the change in predicted probability associated with unit change in an explanatory variable. Therefore, an approximate of the non-linear relationship using marginal effect approximately restores the mechanism of the decomposition framework in Equations 3-5. The linear approximation of the non-linear estimations is given by Equation 5, where μ is the error generated by the linear approximation used to obtain the marginal effects.

### Data

Data for this study were obtained from a prospective cohort study (henceforth referred to as 'the Good Start study') from three diverse sites in South Africa. The background of this study and the selection of sites are described below.

### Context and sites

The aim of the Good Start study was to determine the impact of a PMTCT programme on vertical transmission of HIV. The Good Start study had three sites that were purposively selected. These sites represented a variety of settings that exist in South Africa in three respects: area of residence, antenatal HIV prevalence and health systems functioning [35]. Site A is a peri-urban farm area that had an antenatal HIV prevalence of 9% at the start of the study. Site B is a rural area in one of the poorest regions of South Africa, with a poorly resourced health system and which had an antenatal HIV prevalence of 28% at the start of the study. Site C is a peri-urban township area with a moderately well-resourced health system compared to the other two sites and which had an antenatal HIV prevalence of 47%.

**Figure 1:**
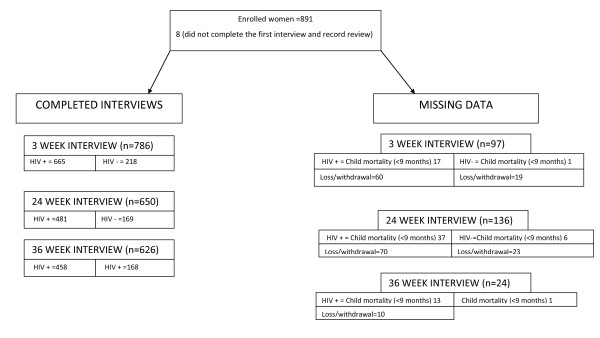
**Study profile**. The left hand side of the figure above indicates all completed interviews at different interviewing periods. The positive and negative sign (+/-) indicates HIV positive and negative women respectively. The right hand side of the figure indicates all missing data which was a result of either mother moving or withdrawal from the study and child mortality.

We followed the enrolled mother-child pairs from delivery to 9 months of age. We measured the HIV status of the infant at 3-4 weeks, 24 weeks and 36 weeks. Of the 891 women initially enrolled in the study, we obtained complete follow-up data for 701 mother-child pairs (78.7%), including 75 (8.5%) child mortality (<9 months) (Figure 1). Doherty [36] has published full details of the data-collection protocol.

The socio-economic position data was collected at 3 weeks and was not done during the first interview. Therefore, we do not have data on socio-economic position for 97 of mother-child pairs at this stage. For the remaining outcomes of interest (immunisation coverage and HIV transmission), we have data on the socio-economic position for 133 and 113 respondents respectively. Table 1 contains the profile of missing data on the above-mentioned outcomes by socio-economic position.

**Table 1:** Mother and child pairs, missing data on immunisation coverage and HIV transmission by socioeconomic position.

	Total number of observations with missing data	Socioeconomic position quintiles				
Child health outcomes	N	Most poor	Very poor	Poor	Less poor	Least poor
Immunisation	133	28(21%)	33(25%)	32(24%)	20(15%)	20(15%)
HIV transmission	113	63(56%)	51(45%)	54(48%)	52(46%)	57(50%)

### Data analysis

We did not restrict the analysis to children that had complete data for all variables. Instead, it was done separately for each variable. Consequently, the number of children with missing data varies through the results.

We carried out the analysis using Stata version 9 [37] and exploratory data analysis using frequency tables. We summarised numerical and categorical data using the appropriate descriptive statistics. We used the Shapiro-Wilk test and histograms to detect departures from normality, and correlation analysis to assess linear associations between numerical variables. We calculated Pearson's product moment correlation coefficient for normally distributed data and Spearman's rank correlation for skewed data. Statistical significance was determined at 5% level.

We included the following explanatory variables: the asset index (our chosen measure of socio-economic position), marital status, site, mother's education, income, mother's age and mother's viral load (only in the case of the HIV transmission outcome). Jackson et al. [36] identified maternal viral load as the single most important factor associated with HIV transmission or death. Therefore, we controlled for maternal viral load at 3 and 36 weeks for the HIV outcome.

## Results

### Summary statistics

Table 2 shows the summary statistics for all the variables. Each socio-economic position quintile had about 20% of households in this population. 81% (123/151) of infants (<9 months) from the most poor households were from the rural site. The least poor quintile largely comprised of households from the peri-urban township [69% (103/150)]. The household monthly income varied significantly across the three sites, but the average monthly household income was R735.

**Table 2:** Basic socio-demographic characteristics of mother and child pairs

Variables	Peri-urban farm area	Rural area	Peri-urban township area	p-value	Combined (N)
Household socioeconomic position					
**Most poor**	21	123	7	0.000***	151
Very poor	33	55	64	0.000***	152
Poor	40	27	83	0.000***	150
Less poor	50	14	87	0.000***	151
Least poor	45	2	103	0.000***	150
Observations (N)	189	221	344		754
Maternal education					
**Primary**	10	13	16	0.483	39
Secondary	156	209	248	0.000***	613
Matric	34	37	143	0.000***	214
Observations (N)	200	259	407		866
Maternal age					
**≤20**	36	87	68	0.000***	191
21-30	130	151	264	0.120	545
>30	34	27	81	0.005***	142
Observations (N)	200	265	413		878
Marital status					
**Married**	40	101	117	0.000***	158
Observations (N)	200	263	408		871
Infant's sex					
Female	110	143	196	0.093*	449
Observations (N)	198	262	414		874
Household income					
Median (R/month)	800	640	700	0.001***	735
Interquartile range	(600-1300)	(320-860)	(400-1200)		(0-1200)
Observations (N)	176	222	299		697

*** p < 0.01, ** p < 0.05, * p < 0.1

Reference categories for categorical variables used in the regression model in bold

71% (613/866) of the women in this study had completed secondary school. 21% (191/878) of the women were below 21 years of age, of which 46% (87/191) were from the rural site. 18% (158/871) of the women in this study were married.

### Principal component analysis

The variables with the highest scores represented indicators of socio-economic position and child health outcomes. A fridge had the highest score of 0.382 followed by water (0.379), a television (0.368), fuel (0.356) and a toilet (0.358) (Table 3).

**Table 3:** Scoring factors and percentage of households owning/using assets in the most poor and the least poor household quintiles

Asset index items	Scoring factors	% of distribution in the sample	Most poor (%)	Least poor (%)
Refrigerator	0.382	48.4	0	100
Radio	0.231	65.8	41.1	91
Television	0.368	47.5	0	40
Stove	0.357	59.2	0	98
Phone	0.329	43.7	0	40
Car	0.161	7.3	0	21
Drinking water (Piped inside the house)	0.379	45.4	0	55
Type of toilet ( Flush toilet)	0.358	48.2	0	41
Cooking fuel (Using electricity for cooking)	0.36	35.4	0	43

Each variable was a binary variable taking a value of 1 if true, and 0 otherwise. The scoring factor is the weight assigned to each variable.

65.8% of the sample owned a radio (Table 3). This was followed by stove ownership at 59.2%, while 48.4% of the sample owned a refrigerator. The least common assets were mainly indicative of the infrastructure at the community level, for example, households with a flush toilet (48.2%), using electricity for cooking (45.4%) and with piped water inside the house (35.4%). A car was the least common of all assets (7.3%).

**Figure 2:**
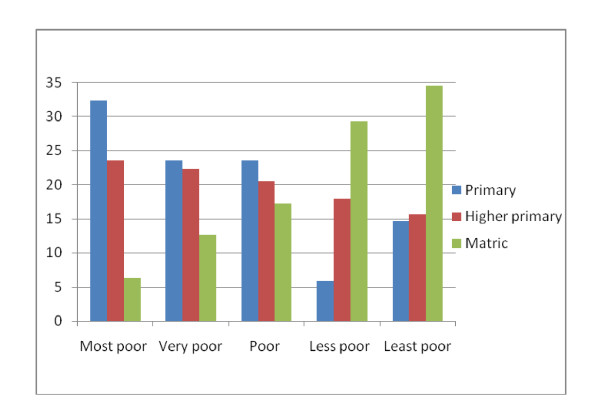
**Wealth quintiles and education**. Primary- no education and successful completion of standard 1&2. Secondary - successful completion standard 3-9. Matric-successful completion of the last year of school.

The asset index produced sharp differences in all assets between the most poor and the least poor quintiles (Table 2). The asset index agreed with the other measures of socio-economic status including education, the food inventory index and income. Similarly, the food inventory index produced expected differences in both basic food items and luxuries (Table 4). Only 6.2% of the most poor had completed high school (matric) compared to 35% of the least poor, whereas 32% of the most poor had primary education as their last standard passed compared to 14.5% of the least poor (Figure 2).

**Table 4:** Percentage of households possessing food items in the most poor and the least poor quintiles

Food possession	Most poor (%)	Least poor (%)
Samp	13.3	64.6
Beans	18.1	97.7
Flour	28.4	86.7
Maize-meal	69.5	97.6
Soup	23.6	96.9
Oil	62.6	99.2
Meat	7.8	96.1
Vegetables	44.1	100
Fruit	8.7	79.7
Rice	48.8	100
Tea	55.9	100
Milk	8.6	89.8
Sugar	0	100
Eggs	11.0	82.0

The food inventory index had a positive relationship with the asset index. The Spearman rank correlation coefficient was 0.30 (p-value 0.000, p < 0.05). We also correlated the asset index with income, which yielded a Spearman rank correlation coefficient of 0.42 (p-value 0.000, p < 0.05) (data not shown). Income had a stronger correlation with the asset index than the food inventory index.

### Marginal effects of determinants

Table 5 shows the marginal effects and the significance of each determinant of the three child health outcomes. We obtained the marginal effects by running regressions of determinants on observed probabilities of each outcome based on Equation 5. The marginal effects demonstrate associations between determinants and health outcomes. Those with positive signs indicate positive associations with the probability of reporting a health outcome, while negative signs indicate negative associations. In addition, the larger the absolute value of a marginal effect, more substantial the association.

**Table 5:** Probability of determinants on reporting health outcome variables

Determinants	Infant mortality (<9 months)	HIV transmission	Immunisation at 24 weeks
Household socioeconomic position			
Very poor	0.0173	0.0144	0.00708
	(0.0318)	(0.0696)	(0.0608)
Poor	0.00835	0.00536	-0.0431
	(0.0351)	(0.0761)	(0.0684)
Less poor	0.0124	0.00404	0.0708
	(0.0369)	(0.0775)	(0.0679)
Least poor	-0.00415	-0.0322	0.0284
	(0.0382)	(0.0816)	(0.0761)
Maternal education			
Secondary	0.0620	-0.0164	-0.0975
	(0.0390)	(0.0738)	(0.0743)
Matric	0.124	-0.0209	-0.0613
	(0.0952)	(0.0819)	(0.0911)
Marital status			
married	-0.0172	-0.0852*	0.0368
	(0.0216)	(0.0511)	(0.0523)
Site			
Rural area	0.0908**	0.227***	-0.434***
	(0.0419)	(0.0779)	(0.0587)
Peri urban township area	0.0252	0.0775	-0.211***
	(0.0276)	(0.0556)	(0.0537)
Maternal age			
21-30	0.00570	-0.0447	0.0866*
	(0.0234)	(0.0601)	(0.0506)
>30	-0.00321	-0.0360	0.0335
	(0.0316)	(0.0686)	(0.0625)
Household income			
(R/month)	-2.25e-06	2.83e-05	-1.92e-06
	(6.73e-06)	(2.32e-05)	(1.53e-05)
Observations	692	432	642

Robust standard errors in parentheses

*** p < 0.01, ** p < 0.05, * p < 0.1

The marginal effects demonstrate associations between explanatory variables and health outcomes. Those with positive signs indicate positive associations with the probability of child mortality/complete immunisation at 24 weeks/HIV transmission. The negative signs indicate negative associations. Furthermore, the larger the absolute value of a marginal effect, more substantial is the association. Reference groups were most poor households, primary education, non-married women, peri-urban farm area and women aged < = 20.

Infants residing in the rural site had a significant positive association with a probability of child mortality. All the other explanatory variables had insignificant associations.

Infants residing in the rural and peri-urban township sites had a reduced probability of completing their immunisations at 24 weeks compared to infants in the peri-urban farm site. Infants of older women (women aged 21-30 years of age) had an increased probability of completing their immunisation at 24 weeks compared to infants of younger women (women aged ≤20).

The rural site had a significant positive association with HIV transmission. In addition, marital status was negatively associated with HIV transmission.

### Socioeconomic position quintiles and child health outcomes

**Figure 3:**
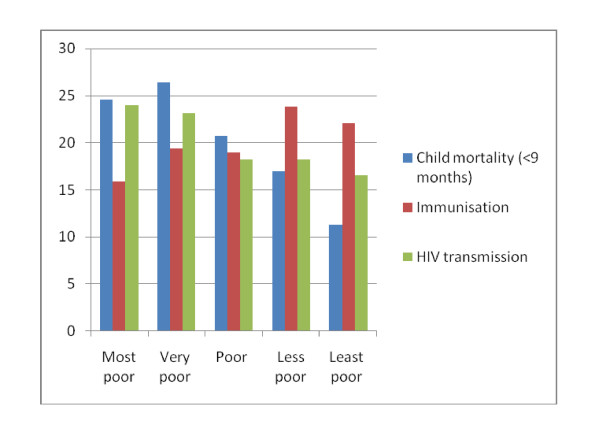
**Wealth quintiles and child health outcomes**.

Dividing the data into asset quintiles clearly indicates inequalities in all child health outcomes. Figure 3 illustrates these relationships and they are described in more detail below. Infant deaths decreased with increasing socio-economic status. One-quarter of the deaths were amongst the most poor compared to 11% in the least poor category. The observed trend of inequalities on the distribution of child mortality (<9 months) approached the 5% significance level (p-value 0.06 and p > 0.05).

HIV transmission at 36 weeks decreased with increasing socio-economic status. The observed inequality in HIV transmission was also marginally significant at p = 0.05. The proportion of all infected children was 24% among the most poor and 17% among the least poor.

Immunisation coverage increased with increasing socio-economic status. Only 15.9% of all completely immunised children were in the poorest quintile at 24 weeks compared to 22.3% of the least poor. The observed inequality trend was statistically significant at p = 0.00 and p < 0.05.

### Concentration curve and index, and child health outcomes

The concentration index analyses further strengthened the above observation of inequality in child health for all three indicators, although only immunisation coverage was statistically significant (Table 6). For child mortality (<9 months), we found a concentration index of -0.088 (p = 0.278 and p > 0.05), indicating a pro-poor inequality, i.e. the data show that infant mortality is higher among the poorest of the population. HIV transmission had a pro-poor concentration index of -0.040 (p = 0.431 and p > 0.05) after controlling for viral load. Immunisation coverage had a statistically significant concentration index inequality of 0.090 (p = 0.000 and p < 0.05) in favour of the least poor households.

**Table 6:** Concentration indices for child health outcomes

Child health outcomes	Concentration indices	p-value
Infant mortality (<9 months)	-0.088	0.278
Immunisation at 24 wks	0.090	0.000*
HIV transmission	-0.040	0.362

*** p < 0.01, ** p < 0.05, * p < 0.1

**Figure 4:**
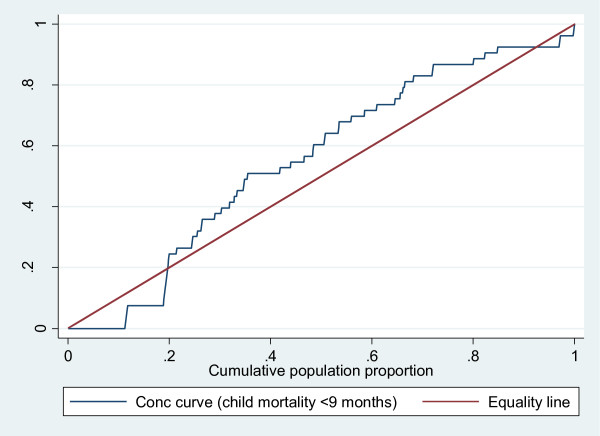
**Concentration curve for child mortality <9 months**.

**Figure 5:**
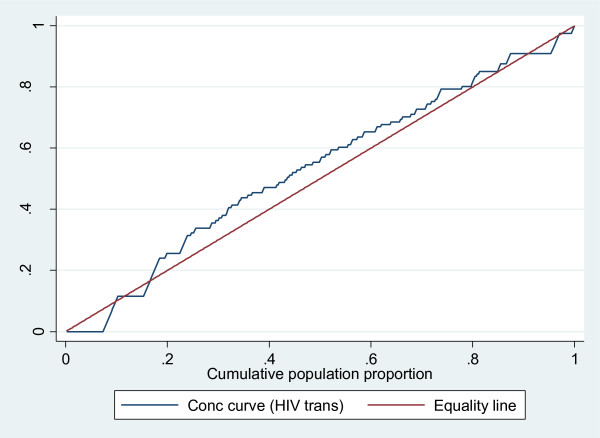
**Concentration curve for HIV transmission**.

**Figure 6:**
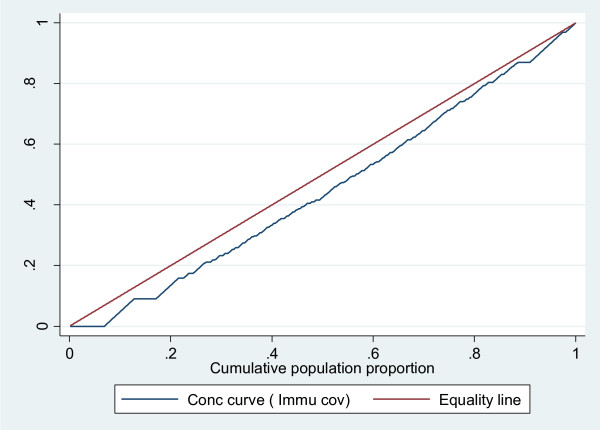
**Concentration curve for Immunisation coverage**.

The concentration curve which plots cumulative percentage of the health variable (y-axis) against the cumulative percentage of the population, ranked by living standards, beginning with poorest, and ending with richest (x-axis). Plots in Figure 4, 5 and 6 confirm the above findings. Both infant mortality and HIV transmission lie above the diagonal (line of equality), which demonstrates that these two outcomes are concentrated amongst the poor. Complete immunisation coverage at 24 weeks lies, below the diagonal, which means this outcome is concentrated amongst the least poor. Figure 4 and 5 also show that the observed inequality in infant mortality and HIV transmission was not statistically significant. This is seen by the concentration curve crossing the diagonal.

### Decomposition of socioeconomic inequality in child health outcomes

Tables 7, 8, 9 are by products of how the marginal effects, mean and concentration indices of determinants translate into absolute contributions to the total observed socioeconomic inequality in health. We observed the absolute contribution of each determinant (Column 3 in Tables 7, 8, 9) by multiplying its marginal effect by the mean of the child health outcome. We can interpret positive (negative) contributions of determinants as that the total health inequality would, other things being equal, be lower (higher) if that determinant had no impact on the child health outcome (instead of that reflected in marginal effects, Table 5) or was equally distributed across the socio-economic spectrum (instead of concentrated, as mirrored in the concentration indices of determinants, Column 2). The analyses reveal that many of the inequalities can be explained by areas of residence (Tables 7, 8, 9).

**Table 7:** Decomposition results Infant mortality (< 9 months)

(Column 1)	(Column 2)	(Column 3)
Determinant	Concentration index (C*_k_*)	Deterministic contribution C -0.088
Household socioeconomic position		
Very poor	-0.3710	-0.0292
Poor	0.0362	0.0018
Less poor	0.4389	0.0229
Least poor	0.8190	0.0085
	***Subtotal***	0.0039
Maternal education		
Secondary	-0.0846	-0.0382
Matric	0.3097	0.0732
	***Subtotal***	0.0350
Maternal age		
21-30	0.0311	0.0038
>30	0.1050	0.0023
	***Subtotal***	0.0061
Site		
Rietvlei	-0.5044	-0.1728
Umlazi	0.2702	0.0230
	***Subtotal***	-0.1498
Marital status		
Married	-0.1900	0.0056
	***Subtotal***	0.0056
Household income		
(R/month)	0.2089	-0.0009
	***Subtotal***	-0.0009
**Residual (unexplained)**		**0.0120**

**Table 8:** Decomposition results HIV transmission

(Column 1)	(Column 2)	(Column 3)
Determinant	Concentration index (C*_k_*)	Deterministic contribution C -0.040
Household socioeconomic position		
Very poor	-0.395	-0.016
Poor	0.021	0.001
Less poor	0.436	0.017
Least poor	0.821	0.039
	***Subtotal***	0.041
Maternal education		
Secondary	-0.081	0.020
Matric	0.284	-0.030
	***Subtotal***	-0.010
Maternal age		
21-30	0.025	-0.006
>30	0.021	-0.002
	***Subtotal***	-0.008
Site		
Rietvlei	-0.557	-0.140
Umlazi	0.235	0.035
	***Subtotal***	-0.105
Maternal viral load		
log viral load at 3 weeks	-0.001	-0.001
log viral load at 36 weeks	0.010	0.002
	***Subtotal***	0.001
Marital status		
Married	-0.199	0.010
	***Subtotal***	0.010
Household income		
(R/month)	0.205	0.016
	***Subtotal***	0.016
**Residual (unexplained)**		**0.016**

**Table 9:** Decomposition results immunisation coverage at 24 weeks

(Column 1)	(Column 2)	(Column 3)
Determinant	Concentration index (C*_k_*)	Deterministic contribution C 0.090
Household socioeconomic position		
Very poor	-0.3382	-0.0004
Poor	0.0667	-0.0009
Less poor	0.4634	0.0077
Least poor	0.8309	0.0048
	***Subtotal***	0.0113
Maternal education		
Secondary	-0.0806	0.0083
Matric	0.3073	-0.0045
	***Subtotal***	0.0038
Maternal age		
21-30	0.0278	0.0025
>30	0.1147	0.0008
	***Subtotal***	0.0033
Site		
Rietvlei	-0.4813	0.1027
Umlazi	0.2858	-0.0282
	***Subtotal***	0.0745
Marital status		
Married	-0.1728	-0.0019
	***Subtotal***	-0.0019
Household income		
(R/month)	0.2047	-0.0013
	***Subtotal***	-0.0013
**Residual unexplained**		0.0005

Two variables: rural and urban township site, and education, made sizeable contributions to the inequality. The rural site accounts for most of the inequality in child health with a contribution of -0.172 (Table 7). Therefore, child mortality (<9 months) as a health outcome is sensitive to the socio-economic variation in site and education.

The observed inequality in HIV transmission is explained by variations in area of residence, namely rural and urban township socio-economic position.

The rural, urban township area and socio-economic position were driving the observed inequality in complete immunisation at 24 weeks (Table 9).

## Discussion

In South Africa, 13 million of the population lives in first world conditions while 23 million people live in developing country conditions [38]. The inequality in South Africa at a general level is therefore abundantly obvious. Our sample represents a portion of the population living under developing country conditions. A major contribution to this paper is that large differences in socio-economic factors and child health outcomes, as well as utilisation of public health services, are documented and quantified for the least well-off groups of the South African population. This highlights the importance of recognising the heterogeneity of the poor.

Overall, child mortality was unequally distributed towards the most poor. The rural site was the main contributor to this inequality. In our sample, the majority of the most poor resides in this rural site, which is characterised by a severe lack of basic infrastructure. For instance, none of the most poor had piped water in their dwellings compared to 97% of the least poor. Water supply and basic sanitation are still reported to be a serious problem in South Africa [39,40]. Mathee, Joffe & Naidoo [41] estimate more than 9 million people are still in need of a basic water supply in South Africa, while more than 16 million people are in need of sanitation services.

The poorest mothers were most likely to transmit HIV to their infants even after controlling for their own viral load. The urban township and peri-urban farm sites drove this disparity. The rural site did not contribute to the overall socio-economic related inequality in HIV transmission. This disparity was mainly because of unequal distribution of the area of residence by asset index.

Both child mortality (<9 months) and HIV transmission were not statistically significant in any of our measures of inequality. These two variables were also influenced by the feeding method. We could therefore hypothesise that the feeding method was inappropriate across the socio-economic positions.

Appropriately identifying disparities in health outcomes is essential, as it informs policy makers about groups that are in greater need of assistance. Even though the population we studied could have been easily identified as homogenously poor, we have shown that it is not. This has important implications for policies aimed at improving child health outcomes amongst the poor.

Policy makers in this context have to consider the tradeoffs between universal coverage and targeting health interventions. The rationale for targeting is that concentrating on 'target groups' of poor households or individuals will achieve a higher impact from a given poverty-alleviation budget or achieve a given impact at a lower budgetary cost. The main challenge with targeting is choosing the appropriate type of targeting mechanism. Targeting programmes have sometimes been called leaking buckets because people that are not the intended beneficiaries tend to use these programmes more.

The free healthcare that South Africa provides to pregnant women and children less than 6 years of age, uses a combination of targeting methods: self selection and the categorical method. Due to disparities between the private sector and the public sector, the provision of healthcare in the public sector is characterised by long waiting lines, shortages of health personnel and equipment, and questionable quality of care. Therefore, non-poor and working women view public sector healthcare as an inferior product. Therefore, we can expect that there are minimal leakages to this section of the population. However, it is an undesirable mechanism that the poor have no choice but to use the public health programmes because they cannot pay for private healthcare, while those that are less poor opt out because of factors related to the quality of care.

In this paragraph, we will use immunisation coverage to demonstrate the performance of free healthcare for pregnant women and children less than 6 years of age. We chose immunisation coverage for three reasons: it is an indicator of use, it was the only finding that showed statistical significance and its causal pathway does not involve other factors, such as infant feeding methods, which may influence both HIV transmission and child mortality. We can interpret the findings of inequality in immunisation coverage in favour of the least poor and that the inequality is being driven by urban township sites, to mean that the urban poor use public health services more than their rural counterparts. In other words, the free healthcare provided to pregnant women and children under 6 years of age is less vertically effective in rural areas. We can hypothesise that transport costs and opportunity costs are factors behind the lower utilisation rates in rural areas. These two factors deter participation in two ways. Firstly, transport costs have been found to constitute a larger share of patient costs in other health interventions [42]. When the women who access public healthcare incur transport costs, healthcare is no longer free to them. Secondly, the women in the rural area included in this study are expected to fetch firewood and do other work. These duties often compromise their participation in health programmes. These duties are a heavy workload and also involve travelling long distances away from home [43]. While individual targeting and categorical targeting are known to be less costly to the health system than other targeting methods, they could be more costly to the households in a rural setting in terms of transport costs and opportunity costs.

Overwhelmingly, the observed inequalities were driven by site irrespective of their direction. This finding suggests that South Africa is facing geographical challenges in securing more equal health across the country. In addition to the site, the asset index and education were important contributors to these inequalities. The decomposition method used in this study allowed us to assess the relative importance of the different inequalities (from determinants of the variable of interest) in generating inequalities in child health outcomes.

The Good Start study enrolled women in the selected sites who participated in the PMTCT programme. Women who accessed private healthcare or lived outside of the three study sites were excluded. This could potentially introduce selection bias because women who did not seek antenatal care would have been excluded. However, the national antenatal coverage is 92% and the mean number of visits per women is 4 [44]. Given these high utilisation rates, it is not unreasonable to assume that the majority of women in these sites would have had an opportunity to participate in the study. However, it is likely that the remaining 8% of women who did not attend antenatal care could be even more deprived than the women in our study. If so, the results would be a downward bias in estimating the socio-economic position relationship. Therefore, you may choose to consider the results of the study's lower-bound estimates.

The second source of bias resulting from the sampling strategy is the exclusion of relatively wealthy women. Indeed, excluding the wealthier subset of the population, a priori, would bias the sample. This would be important in a national study investigating the overall inequality between the rich and the poor. The exclusion of wealthier women is less relevant for this paper, since our aim was to determine whether inequalities exist among the poor. As a result, in this paper, we refer to the most poor and the least poor to further highlight the absence of the rich.

## Conclusion

The South African government provides free basic healthcare to pregnant women and children under 6 years of age. Despite this, the differences in immunisation coverage suggest that the use of this free healthcare is uneven. This is further supported by the differences found in HIV transmission and mortality. We believe that most of the inequalities observed in this study are avoidable and remediable, and that they are therefore unjust.

In the light of these observations, it appears that child health programmes in South Africa need to be more explicitly designed to reduce inequalities in health. Programme planners should realise that universal rollout strategies are not sufficiently effective in reaching communities that have suffered chronic deprivation and marginalisation. Benefit incidence studies have shown the rich to benefit most from public investments in education and health compared to the poor [45]. Furthermore, economic development translates faster into mortality trend declines among the better-off [3,46]. Thus, it is important to recognise that an underlying lack of basic infrastructural services, such as running water, adequate sanitation and electricity, can undermine child health programmes such as prevention of mother to child transmission of HIV. Therefore, to ensure that children who are in greater need of health interventions access and benefit from health interventions, delivery strategies should specifically target them.

The South African government should consider geographical targeting for future health service provision programmes. The National Community Health Worker (CHW) programme is currently under discussion. This could be an opportunity to reach the most poor as they are in greater need.

## Competing interests

The authors declare that they have no competing interests.

## Authors' contributions

LN conceptualised the research question in discussion with MC, and BJ. LN analysed the data. The results of the analysis were discussed and agreed upon by all authors. LN wrote the first draft of the paper. All authors contributed to and refined subsequent drafts. All authors read and approved the final manuscript.
